# Semi-quantitative metabolic values on FDG PET/CT including extracardiac sites of disease as a predictor of treatment course in patients with cardiac sarcoidosis

**DOI:** 10.1186/s13550-017-0315-y

**Published:** 2017-08-18

**Authors:** Mitsutomi Ishiyama, Laurie A. Soine, Hubert J. Vesselle

**Affiliations:** Department of Radiology, University of Washington, 1959 NE Pacific Street, Seattle, WA 98195 USA

**Keywords:** Sarcoidosis, Cardiac sarcoidosis, FDG, PET/CT, Maximum SUV, Semi-quantitative value

## Abstract

**Background:**

Cardiac sarcoidosis is associated with major adverse cardiac events including cardiac arrest, for which anti-inflammatory treatment is indicated. Oral corticosteroid is the mainstay among treatment options; however, adverse effects are a major concern with long-term use. It would be beneficial for providers to predict treatment response and prognosis for proper management strategy of sarcoidosis, though it remains challenging. Fluorine (F)-18 fluorodeoxyglucose (FDG)-positron emission tomography(PET)/computed tomography(CT) has an advantage over anatomical imaging in providing semi-quantitative functional parameters such as standard uptake value (SUV), metabolic volume, and total lesion glycolysis (TLG), which are well-established biomarkers in oncology. However, the relationship between these parameters and treatment response has not been fully investigated in cardiac sarcoidosis. Also, the prognostic value of extracardiac active inflammation noted on FDG-PET/CT in the setting of cardiac sarcoidosis is unclear. The aim of this retrospective study was to investigate the prognostic value of semi-quantitative values of both cardiac and extracardiac disease sites derived from FDG-PET/CT in predicting treatment course in cardiac sarcoidosis.

**Methods:**

Sixteen consecutive patients with suspected cardiac sarcoidosis, who demonstrated abnormal myocardial activity on cardiac-inflammation FDG-PET/CT encompassing the entire chest/upper abdomen and subsequently underwent corticosteroid therapy for diagnosis of active cardiac sarcoidosis, were included. Semi-quantitative values of hypermetabolic lesions were derived from all visualized organ system and were compared to daily corticosteroid dose at 6 months.

**Results:**

Of the 16 patients, 81.3% (13/16) of the patients showed extracardiac involvement. The lesion with the greatest SUV was identified in the heart in 11 patients (68.7%), in the liver in 1 patient (6.3%), and in lymph nodes in 4 patients (25%). The maximum SUV across all visualized organ systems including the heart were 8.8 ± 3.1 for the patients with corticosteroid dose ≤ 10 mg and 12.5 ± 3.3 for those with > 10 mg (*P* = 0.04). Metabolic volume and TLG across all visualized organ systems or any values in the heart alone showed no significant statistical difference between the two groups.

**Conclusions:**

Maximum SUV across all involved organ-systems of the chest and upper abdomen, not that of the heart alone, could be a predictor of treatment course of steroid therapy at 6 months in patients with active cardiac sarcoidosis.

## Background

Sarcoidosis is an idiopathic multisystem inflammatory granulomatous disease, for which various environmental causes have been proposed in relation to its development, but not yet elucidated [1]. It frequently involves the lungs, eyes, lymph nodes, and skin; however, many of patients do not require treatment until they develop disability or impaired organ function. While cardiac involvement has historically thought to be quite rare, autopsy studies suggest that about 25% of sarcoidosis patients have cardiac involvement [1]. Although approximately 5% of sarcoidosis patients present with cardiac symptoms, anti-inflammatory treatment is generally recommended due to high risk ventricular arrhythmias in this population [1].

Oral corticosteroid is the mainstay among treatment options; however, adverse effects are a major concern with long-term use. Predicting treatment response or prognosis would be beneficial to healthcare providers managing sarcoidosis patients. However, assessing the inflammatory activity of sarcoidosis remains challenging despite current multimodality imaging and laboratory studies.

F-18 FDG PET/CT has emerged as a potentially useful modality for the evaluation of inflammatory diseases such as sarcoidosis [2–17]. FDG PET/CT has been shown to be of superior diagnostic ability when compared to Gallium-67 scintigraphy, the conventional assessment tool for sarcoidosis inflammation. The main advantage of FDG PET/CT is its ability to demonstrate the metabolic activity as opposed to anatomic imaging such as radiography, CT and magnetic resonance imaging (MRI), which are unable to differentiate an active focus from inactive or fibrotic changes. Moreover, FDG PET/CT allows for the semi-quantification of metabolic activity with standard uptake value (SUV), metabolic volume, or total lesion glycolysis (TLG), which have been shown useful in predicting prognosis or treatment response in oncology. To date, there have been encouraging reports about the usefulness of FDG PET/CT in the evaluation of sarcoidosis regarding to the extent and activity of inflammation [4, 8, 9, 11, 15–17], response assessment [4, 5, 7, 9], and prognostic assessment [6, 10, 12]. However, the relationship between semi-quantification of FDG uptake and treatment response and prognosis has not been fully investigated. Also, the prognostic value of extracardiac active inflammation noted on FDG-PET/CT in the setting of cardiac sarcoidosis is unclear.

The aim of this study was to investigate the prognostic significance of FDG uptake in both cardiac and extracardiac disease sites as measured by semi-quantitative values derived from F-18 FDG PET/CT (maximum SUV, metabolic volume, and TLG), in predicting treatment course in patients with cardiac sarcoidosis.

## Methods

### Patient

The University of Washington Institutional Review Board approved this retrospective study and the need for consent was waived. Between January 2009 and May 2015, 159 patients underwent dedicated cardiac-inflammation F-18 FDG PET/CT for suspected cardiac sarcoidosis. We excluded patients with no abnormal FDG activity in the heart (*n* = 77), abnormal FDG activity in the heart but insufficient clinical evidence for medical intervention (*n* = 16), abnormal FDG activity in the heart and treatment corticosteroid treatment was initiated, but treatment was discontinued due to steroid induced myopathy (*n* = 1), abnormal FDG activity in the heart and treatment was initiated, but the regimen included both corticosteroid and immunomodulating agents (*n* = 7), suboptimal imaging quality due to incomplete suppression of physiologic cardiac FDG uptake (*n* = 15; 5.7% of the total population), and no medical record available (*n* = 27). Finally, we identified 16 patients (M:4, F:12) with average age of 50.3 years old (range 33–77 years) who demonstrated abnormal myocardial FDG uptake on dedicated cardiac-inflammation F-18 FDG PET/CT, and subsequently underwent corticosteroid therapy for a diagnosis of active cardiac sarcoidosis (Table 1).

**Table 1:** Study population demographics, overall treatment course with daily oral prednisone, and follow-up modalities

Patient no.	Sex	Age	Pathological evidence of extracardiac sarcoidosis (Y/N)	Daily corticosteroid dose and tapering protocol				Initial evaluation		3-month visit evaluation			6-month visit evaluation		
				Initial dose	Dose at 3-month visit	Dose at 6-month visit	Dose modification at 9-month visit	Pulmonary function test	Echocardiography	Pulmonary function test	Echocardiography	PET/CT	Pulmonary function test	Echocardiography	PET/CT
1	M	50	Y	40 mg x 2 weeks, tapered to 20 mg	20 mg	20 mg	No change	Y	Y	Y	Y	Y	Y	N	Y
2	F	49	Y	40 mg x 2 weeks, tapered to 20 mg	20 mg	7.5 mg	No change	Y	Y	N	N	Y	N	Y	Y
3	M	35	Y	40 mg	20 mg	0 mg	No change	Y	Y	Y	Y	Y	N	Y	Y
4	M	61	Y	20 mg x 4 weeks, tapered to 10 mg	15 mg	5 mg	No change	Y	Y	Y	N	Y	Y	N	Y
5	M	57	N	40 mg x 2 weeks, tapered to 20 mg	20 mg	25 mg	No change	Y	Y	Y	Y	Y	Y	N	Y
6	M	45	N	40 mg x 2 weeks, tapered to 20 mg	10 mg	10 mg	No change	Y	N	Y	N	N	N	Y	N
7	M	77	Y	20 mg	0 mg	0 mg	No change	Y	N	N	N	N	Y	N	N
8	M	38	N	40 mg x 2 weeks, tapered to 20 mg	10 mg	30mg x 4 weeks, tapered to 20 mg	Increased to 40 mg	Y	N	Y	Y	Y	Y	Y	Y
9	F	63	Y	60 mg	20 mg	5 mg	No change	N	N	N	N	Y	N	N	Y
10	M	48	Y	60 mg	20 mg	10 mg	No change	Y	Y	N	N	N	N	Y	Y
11	M	49	Y	40 mg x 4 weeks, tapered to 25 mg	20 mg	10 mg	Decreased to 5 mg	Y	N	Y	N	Y	Y	N	Y
12	M	61	Y	40 mg x 2 weeks, tapered to 20 mg	40 mg x 4 weeks, tapered to 30 mg	15 mg	No change	Y	N	N	N	Y	Y	N	N
13	F	46	N	30 mg x 4 weeks, tapered to 20 mg	40 mg x 4 weeks, tapered to 25 mg	15 mg	No change	Y	Y	Y	N	Y	N	N	N
14	F	33	Y	40 mg x 4 weeks, tapered to 20 mg	10 mg	10 mg	No change	Y	Y	N	Y	Y	Y	N	N
15	M	49	N	20 mg	20 mg	5 mg	Decreased to 2.5 mg	Y	N	Y	Y	Y	Y	N	Y
16	M	44	N	40 mg x 2 weeks, tapered to 20 mg	20 mg	10 mg	No change	Y	Y	Y	N	N	Y	N	N

### Diagnosis

Sarcoidosis specialists made diagnosis of cardiac sarcoidosis. The diagnosis was based on a combination of pathological confirmation of extracardiac involvement, cardiac studies (electrocardiogram and cardiac ultrasound), and positive cardiac findings on F-18 FDG PET/CT combined with concurrent cardiac perfusion PET/CT with or without other radiological studies including cardiac MRI in 10 patients. In six patients, the diagnosis was made based on clinical history, cardiac study, and positive cardiac findings on F-18 FDG PET/CT combined with concurrent cardiac perfusion PET/CT with or without other radiological studies including cardiac MRI, but without pathological confirmation. Seven patients underwent cardiac MRI within 3 months of PET/CT prior to treatment, and all of them showed positive late gadolinium enhancement corresponding to hypermetabolic foci on FDG PET/CT, pattern of which was consistent with sarcoidosis involvement.

Therefore, 10 of 16 patients met the widely accepted diagnostic criteria established by the Japanese Ministry of Health and Welfare [18] or Heart Rhythm Society [19]. Six patients did not meet the criteria because these patients lacked pathological confirmation of cardiac or extracardiac sarcoidosis, though three of them showed positive late gadolinium enhancement on cardiac MRI. These six patients were presumed to have cardiac sarcoidosis by our experienced sarcoidosis experts at the referring center.

### Treatment and follow-up

After the diagnosis of sarcoidosis was established, oral steroid treatment was initiated with 60 mg (*n* = 2), 40 mg (*n* = 10), 30 mg (*n* = 1), or 20 mg (*n* = 3) of daily prednisone based on the severity of symptoms and of cardiac/radiological studies. Steroid therapy was tapered down to 15–25 mg in most of the patients by 3-month follow-up visit. At 3- and 6-month follow-up visits, daily dose of prednisone was modified as shown on Table 1, according to response to the treatment determined by patient interval history, symptoms, and follow-up studies if available, such as PET/CT (12 patients at 3-month and 10 patients at 6-month), pulmonary function test (10 patients at 3- and 6-month each), and cardiac ultrasound (6 patients at 3-month and 5 patients at 6-month) (Table 1).

### Patient groups

The daily oral corticosteroid dose required to control the disease at 6-month was determined and dichotomized into two groups for analyses; either > 10 mg or ≤ 10 mg. In 13 patients, the daily steroid dose prescribed at 6-month visit was considered satisfactory as there was no apparent worsening of the disease for at least the subsequent 3-month and no dose modification was made at the next subsequent evaluation (9-month follow-up visit). Two patients achieved interval dose reduction from 10 to 5 mg and 5 to 2.5 mg, and were classified into the group with a daily dose of ≤ 10 mg with no group change. One patient showed disease progression between 6-month and 9-month visits, and the steroid dose was increased from 20 to 40 mg, and was classified into the group with a daily dose of > 10 mg with no group change.

## F-18 FDG PET/CT protocol

### Patient preparation

Each patient was instructed to follow a no carbohydrate, high-protein diet (specifically meat, eggs, and nuts) with non-sweetened drinks (water, plain coffee, or tea) for the entire day before the study, and to fast for 12 h before the study to shift the myocardium to fatty acid metabolism and suppress myocardial glucose utilization.

### PET/CT acquisition (F-18 FDG PET combined with cardiac perfusion PET)

All the PET studies were performed with a dedicated PET/CT system (Discovery STE; GE Healthcare, Milwaukee, WI, USA) equipped with a 16-detector row helical CT scanner. The sarcoidosis inflammation protocol consisted of both myocardial perfusion PET/CT and FDG PET/CT, as follows:

First, myocardial perfusion PET/CT was performed using either [N-13]-NH_3_ (*n* = 4) or Rb-82 (*n* = 12). 10 min after the perfusion PET scan, unfractionated heparin (50 IU/kg) was injected to further force the myocardium into fatty acid metabolism and was followed by an injection of 259–407 MBq (7–11 mCi) of F-18 FDG. Blood glucose was checked in all patients before the FDG PET study, and all glucose levels were <150 mg/dL.

For the FDG PET/CT, an attenuation correction CT (120 kV, 20–120 mA with automatic exposure control, CT dose index 3.26 mGy) was performed over the heart, and the gated cardiac PET scan was started 50–60 min after FDG injection with a single 12-min per-bed two-dimensional (2D) (*n* = 11) or 8-min per-bed three-dimensional (3D) acquisition (*n* = 5). Subsequently, a non-gated FDG PET of the entire thorax and upper abdomen was performed from the supraclavicular region down to below the liver after an attenuation correction CT scan spanning the same region. The non-gated FDG PET/CT of the thorax and abdomen was performed with either a 7-min per-bed 2D acquisition (*n* = 11) or a 5-min per-bed 3D acquisition (*n* = 5). Attenuation correction PET images were reconstructed with an OSEM (ordered subset expectation maximization iterative) algorithm. The average time interval between the tracer injection and non-gated FDG PET/CT was 74.6 min (61–87 min).

### Image post-processing

All image post-processing was performed on a commercially available workstation (Advantage Windows Workstation, GE Healthcare).

**Fig. 1:**
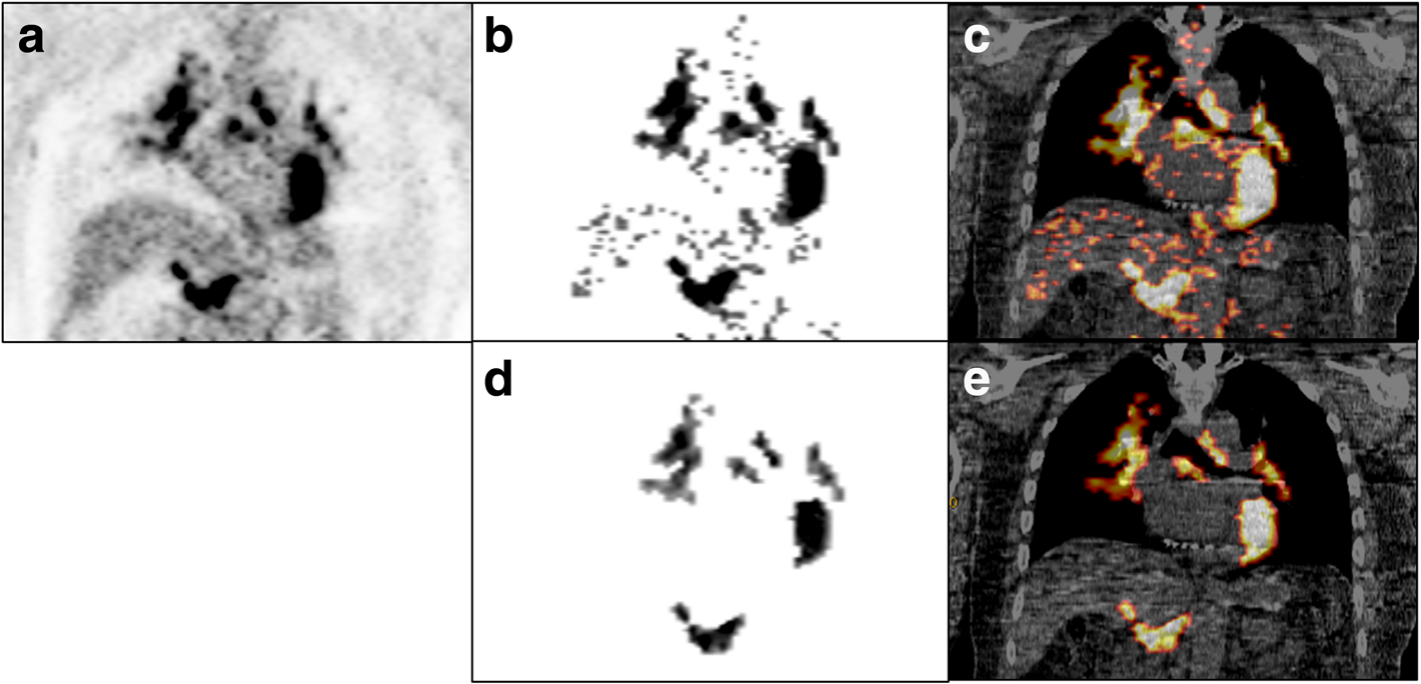
Post-processing of FDG PET/CT images. Attenuation-corrected coronal PET image (**a**), post segmented PET (using a threshold of max SUV of 2.5) (**b**) and fused PET/CT (**c**) images, and post refined PET (**d**) and fused PET/CT (**e**) images

Attenuation-corrected PET images were first segmented using a threshold of maximum SUV of 2.5 and were followed by removal of noise and geometric refinement using the cutting tool on both axial and coronal PET images. This step was done while referring to the attenuation correction CT and PET/CT fusion images as shown in the Fig. 1. A volume-of-interest (VOI) was created for each lesion over the segmented PET images.

Semi-quantitative values (maximum SUV, TLG, and metabolic volume) were derived from all lesions with abnormal FDG uptake in each visualized organ system. SUV was defined as tissue concentration (uCi/ml) divided by administered dose (mCi)/patient weight (Kg). Metabolic volume of the lesion was determined by the entire volume of the VOI. Total lesion glycolysis (g) was calculated as the product of the mean SUV and metabolic volume of the lesion.

### Statistical analysis

Statistical analyses were performed using commercially available statistical software (PASW statistics, version 18.0, SPSS Inc., Chicago, IL, USA). All continuous variables are expressed as the mean ± SD (range). The following statistical analyses were performed:

1. The differences in the semi-quantitative values of all involved organ systems including the heart between the two groups (daily steroid dose of > 10 mg or ≤ 10 mg) using Mann-Whitney *U* test.
2. The differences in the semi-quantitative values of the heart alone between the two groups using Mann-Whitney *U* test.
3. The relationship between the presence or absence of pulmonary, lymph node, or extrathoracic organ system involvement and daily oral steroid dose at 6-month using the Fisher’s exact test.
4. The relationship between the max SUV and metabolic volume of all involved organ systems including the heart, lymph nodes, heart, and lung. This analysis was not performed for extrathoracic organ system (liver, spleen, and bone) separately due to the small number of the patients involved.

## Results

Of the 16 patients, 81.3% (13/16) of the patients showed hypermetabolic extracardiac involvement in lymph nodes, 37.5% (6/16) in lung parenchyma, 18.8% (3/16) in the liver, 25% (4/16) in the spleen, and 18.8% (3/16) in bones. The lesion with the highest SUV was identified in the heart in 11 patients (68.7%), in the liver in 1 patient (6.3%), and in lymph nodes in 4 patients (25%).

**Fig. 2:**
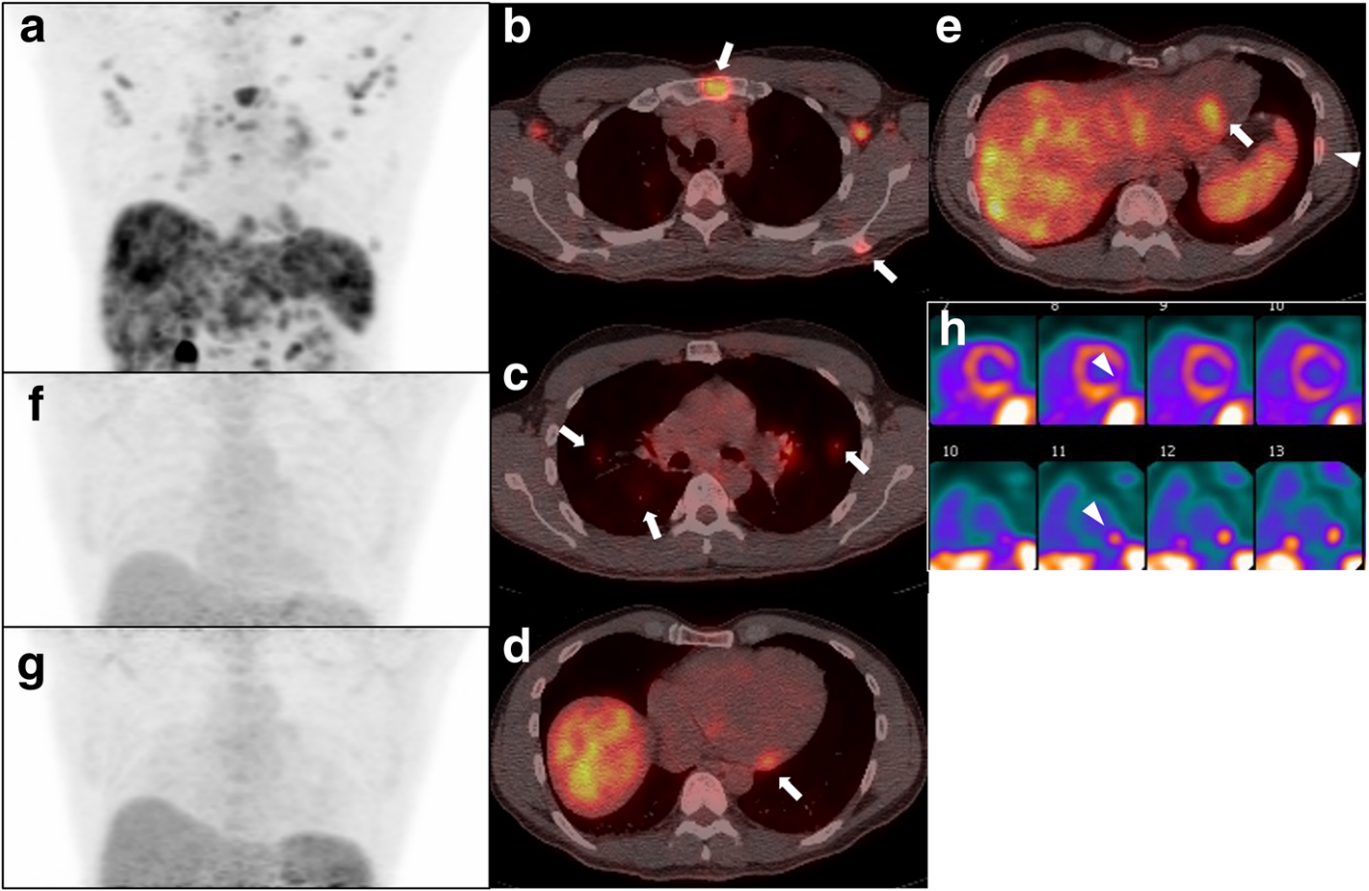
Representative case from the group with daily maintenance dose of prednisone of ≤ 10 mg (patient no. 3). Pretreatment FDG PET/CT (**a** 3D maximum intensity projection; **b**, **c**, **d**, **e** fused PET/CT) demonstrate multiple organ diseases involving lymph nodes (thoracic and abdominal), the lung (**c**, arrows), bones (**e** arrowhead), heart (**d**, **e** arrows), liver, and spleen. Reconstructed short axis images of the left ventricle (**h** perfusion images on the top and FDG images on the bottom) show increased metabolic activity in the inferolateral wall with corresponding mildly reduced perfusion (arrowheads), suggestive of active inflammation. The max SUV, metabolic volume, and total lesion glycolysis of all involved organ system and the heart are 7.5 (liver), 1860.9 ml, and 6866.9 g, and 5.7, 84.1 ml, and 294.4 g, respectively. Follow-up PET/CTs performed at 3 months (**f**) and 9 months (**g**) since the initial study show complete metabolic resolution. The patient initially received 40 mg of corticosteroid daily, which reduced to 0 mg at 6 months

**Fig. 3:**
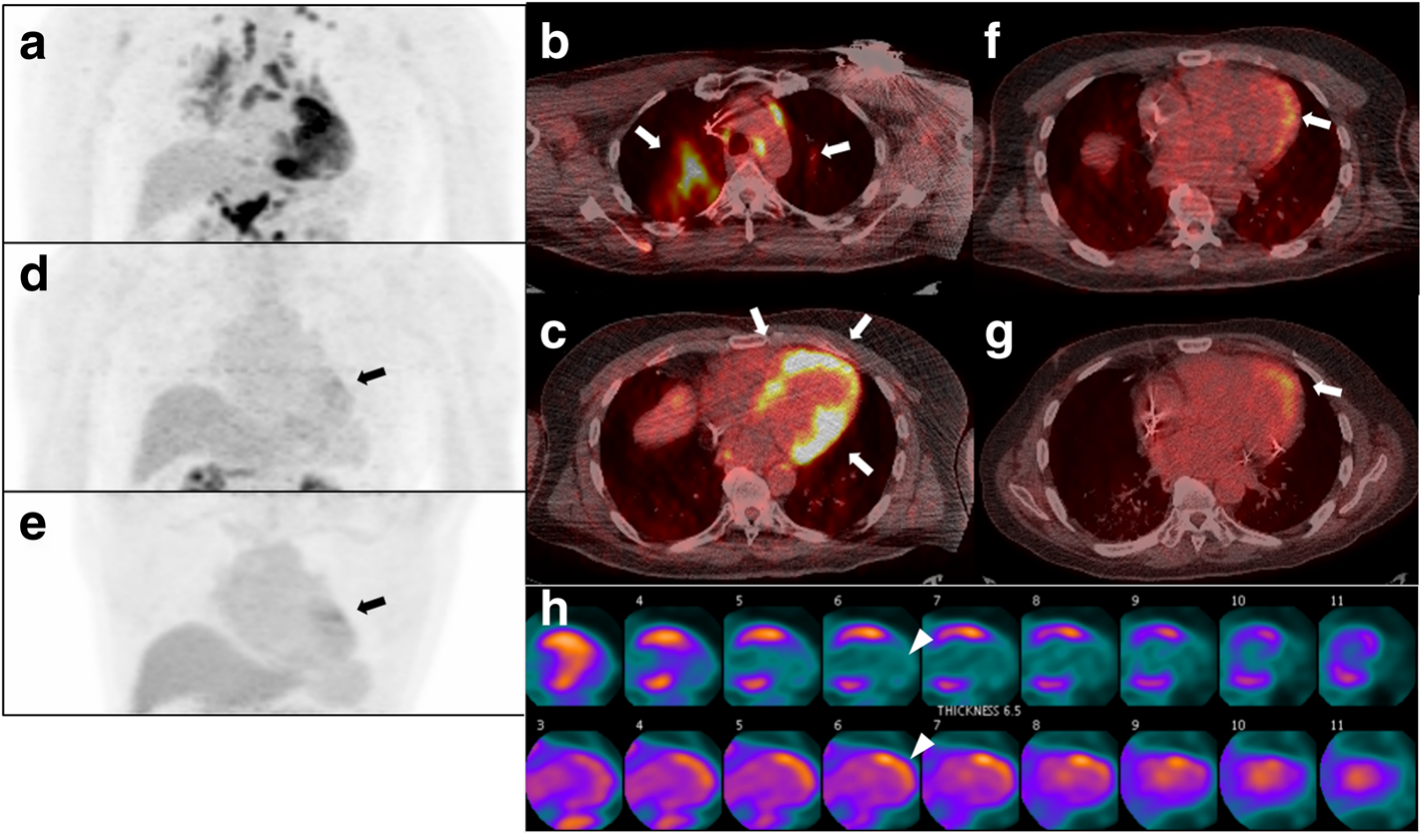
Representative case from the group with daily maintenance dose of prednisone of > 10 mg (patient no. 12). Pretreatment FDG PET/CT (**a** 3D maximum intensity projection; **b**, **c** fused PET/CT) demonstrate multiple organ disease involving lymph nodes (thoracic and abdominal), the lung (**b** arrows), and heart (**c** arrows). The max SUV, metabolic volume, and total lesion glycolysis of all involved organ system and the heart are 15.9 (heart), 580.3 ml, and 2782.5 g, and 15.9, 246 ml, and 1393.9 g, respectively. Follow-up PET/CT performed at 3 months since the initial study (**d**, **f**) show near complete metabolic resolution except for persistent metabolic activity in the heart (**d**, **f** arrows, max SUV 4.0). The patient was started with 30 mg of daily corticosteroid, but the dose was increased to 40 mg at 3 months visit, which was later reduced to 15 mg at 6 months. The patient continued on corticosteroid therapy for years and later methotrexate was added to the treatment regimen, though follow-up PET/CT at 4 years since the initial study still show persistent metabolic activity (**e**, **g** arrows, max SUV 4.1) in the heart. Reconstructed vertical long axis images of the left ventricle at 4 years since the initial study (**h** perfusion images on the top and FDG images on the bottom) show increased metabolic activity in the anterior and anterolateral wall with corresponding reduced perfusion (arrowheads), suggestive of active inflammation. Note that reduced perfusion in the apical 1/2 of the inferior wall without FDG activity is compatible with scar tissue

Twelve patients were classified into a group with a daily oral maintenance dose of prednisone of ≤ 10 mg, and 4 patients were classified into a group with a dose of prednisone of > 10 mg. Representative cases from each group are shown in the Figs. 2 and 3.

**Fig. 4:**
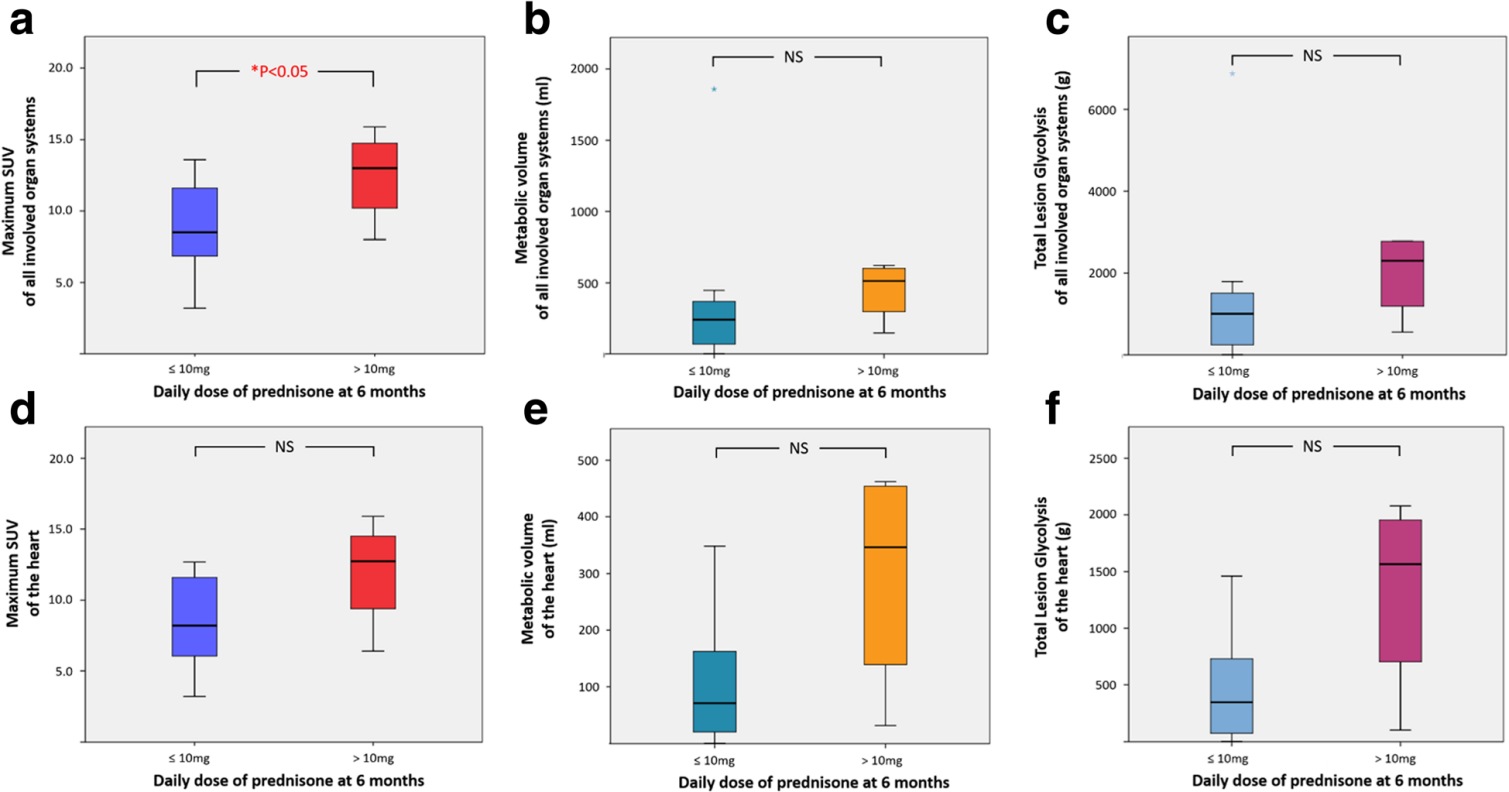
Box plots of differences in the semi-quantitative values of all involved organ systems including the heart (**a**, **b**, **c**) and the heart alone (**d**, **e**, **f**) between patients with daily oral steroid dose of less or equal to 10 mg and those with greater than 10 mg. * represents statistically siginificant difference. The central box represents values from lower to upper quartile (25–75%), the middle line represents the median, vertical bars extend from 5 to 95%, and asterisks represent outliers

The differences in the semi-quantitative values of all involved organ systems and those of the heart between two patient groups are shown in the Table 2 and Fig. 4. There was a statistically significant difference in the maximum SUV among all involved organ systems between the groups, but none of the values in the heart alone showed any significant statistical difference.

**Table 2:** Differences in semi-quantitative values for all involved organ systems including the heart and for those of the heart alone between patients with daily oral steroid dose of less or equal to 10 mg and greater than 10 mg. * represents statistically significant difference

	Organ system	Daily steroid equal to or less than 10 mg	Daily steroid greater than 10 mg	*P* value
All involved organ systems	Max SUV	8.8 ± 3.1	12.5 ± 3.3	* 0.04
	Metabolic volume (ml)	343 ± 501	449 ± 215	0.17
	Total lesion glycolysis (g)	1340 ± 1349	1986 ± 1054	0.13
	Max SUV	8.5 ± 3.1	11.9 ± 4.0	0.078
Heart	Metabolic volume (ml)	108 ± 118	296 ± 202	0.078
	Total lesion glycolysis (g)	473 ± 517	1328 ± 879	0.078

As shown in Table 3, there were no statistically significant differences between the presence or absence of pulmonary, lymph node or extrathoracic organ system involvement (liver, spleen, and bone), and oral steroid dose at 6 months.

**Table 3:** Relationship between the presence or absence of pulmonary, lymph nodes, or extra-thoracic organ system involvement (liver, spleen, and bone) and daily oral steroid dose at 6 months

Organ/system	Presence (+) or absence (-) of involvement	Daily steroid equal to or less than 10 mg	Daily steroid greater than 10 mg	*P* value
Lung	+ (*n* = 6)	4	2	0.60
	− (*n* = 10)	8	2	
Lymph nodes	+ (*n* = 13)	10	3	1.0
	− (*n* = 3)	2	1	
Extrathoracic organ system	+ (*n* = 6)	6	0	0.23
(Liver, spleen, or bone)	− (*n* = 10)	6	4	

**Fig. 5:**
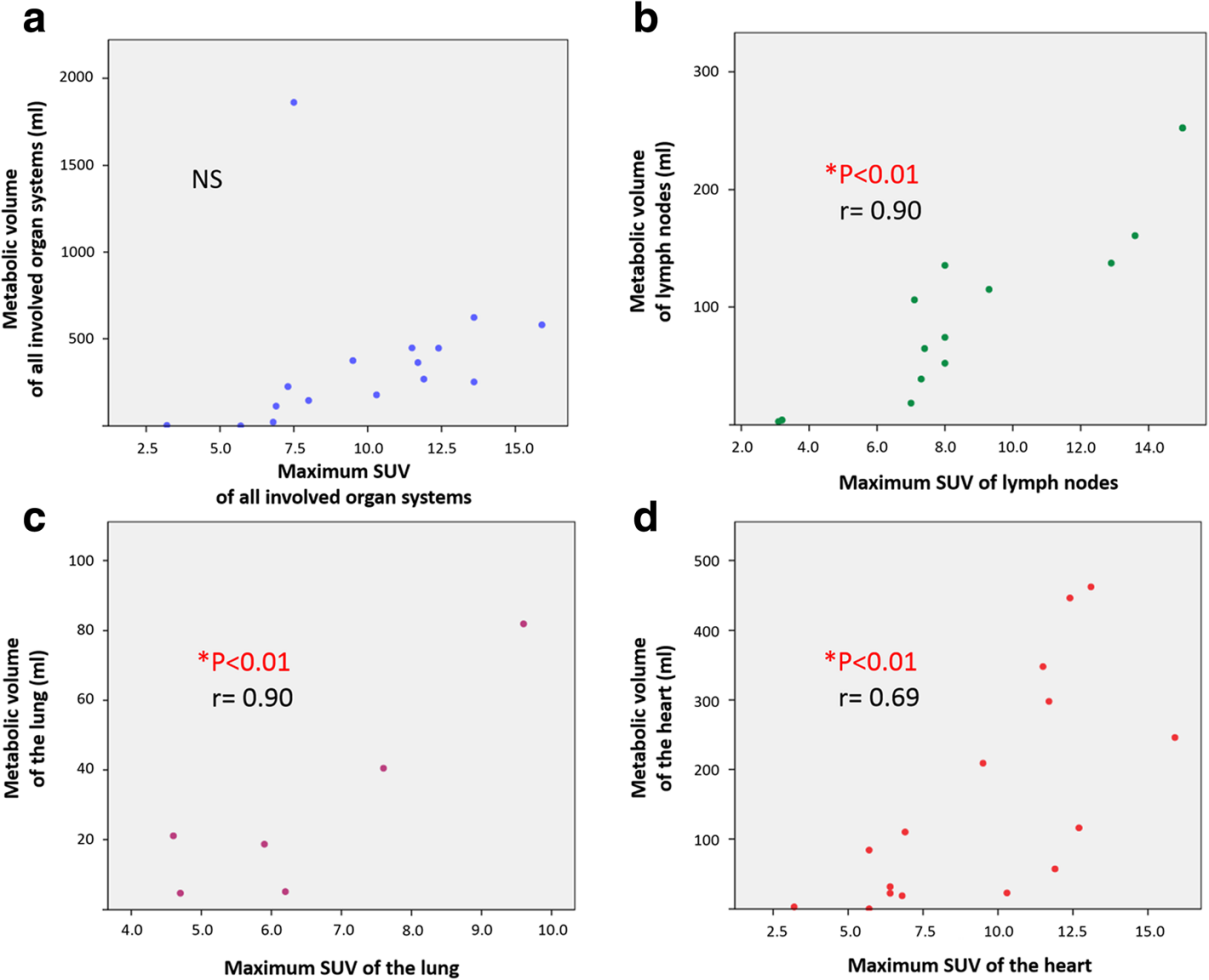
Relationship between the max SUV and metabolic volume of all involved organ systems including the heart (**a**), lymph nodes (**b**), lung (**c**), and heart (**d**). * represents statistically significant difference

The relationships between the max SUV and metabolic volume of all involved organ systems, heart, lung, and lymph nodes were shown in Fig. 5. There were statistically significant relationships between the max SUV and metabolic volume of the heart, lung, and lymph nodes, but not in the values of all involved organ systems.

## Discussion

Maximum SUV across all involved organ systems was a significant predictor of maintenance dose of oral corticosteroid at 6 months following initiation of treatment in patients with cardiac sarcoidosis. Neither metabolic volume nor TLG of all involved organ systems showed a significant relationship with maintenance dose of corticosteroid at 6 months. On the other hand, none of the three semi-quantitative values in the heart predicted corticosteroid dose at 6 months. Maximum SUV of the heart did not reach statistical significance, though it showed weak correlation with daily steroid dose at 6 months. This was attributed to extracardiac organ system involvement with greater maximum SUV than the heart, which was noted in about one third of the patients. Similarly, none of the involved extracardiac organ systems (lung, lymph node, and extrathoracic disease sites) independently demonstrated significant relationship with daily steroid dose at 6 months.

The present study suggests that evaluation of extracardiac organ systems, in addition to the heart, is helpful to estimate the short- to intermediate-term treatment course in patients with active cardiac sarcoidosis. While corticosteroid therapy is the first line treatment option for active sarcoidosis, its adverse effects could limit the benefit of treatment, especially for those patients requiring long-term treatment. Assessment of FDG PET/CT of the thoracic and upper abdomen is beneficial for patients expected to require prolonged treatment by providing prognostic information so that treatment regimen could be tailored, such as early addition of or switch to methotrexate or other immunomodulating therapy, to maximize the patient benefit [1].

FDG PET/CT has been shown to be useful in determining treatment option in patients with sarcoidosis [7, 9, 16], largely due to the detection of metabolically active disease [4, 8, 9, 11, 15–17] and evaluation of treatment response [4, 5, 7, 9]. To date, a few studies have been published evaluating the prognostic value of PET/CT [6, 10, 12]. Keijsers et al. [10] investigated the prognostic value of metabolic activity in the lung parenchyma with 43 newly diagnosed sarcoidosis patients. They found that if untreated, diffuse parenchymal disease on PET/CT, as opposed to parenchymal disease on CT without metabolic activity, predicted future deterioration of diffusion capacity measured by pulmonary function test. Blankstein et al. [6] focused on the cardiac FDG PET/CT findings concurrently performed with Rubidium-82 perfusion PET/CT. They concluded that abnormal FDG activity accompanied by focal perfusion defect on Rubidium-82 PET/CT was a significant indicator of future major cardiac event. These studies primarily investigated patients who were not undergoing corticosteroid or other anti-inflammatory therapy and therefore, their findings would influence the timing of medical intervention, but not the treatment regimen. In contrast, the present study highlights the prognostic value of FDG PET/CT regarding treatment course in patients who are on corticosteroid therapy, and thus helpful in tailoring the treatment regimen. These data synergistically further enhance the value of PET/CT in evaluating patients with clinically active sarcoidosis.

The present study shows that the intensity of sarcoidosis expressed by the maximum SUV is a more significant prognostic indicator than metabolic volume, which reflects the extent of inflammation. Physiologically, this finding appears reasonable as the anti-inflammatory effect of corticosteroid would be expected to be less in highly active inflammatory foci even if small, while a pronounced response would be expected in less active foci even if extensive. Alternatively, it is noteworthy that there was no significant relationship between the intensity and extent of inflammation when all involved organ systems are considered as a whole. This may imply that a patient with extensive disease does not always present with intense disease or vice versa. Moreover, it is even more significant in terms of prognosis when a patient presents with limited but intense disease. Therefore, caution should be exercised during interpretation of FDG PET/CT not to underestimate the amount of corticosteroids needed to successfully treat small volume disease of high intensity.

However, as one may presume, the intensity and extent of the disease are not independent when examining each involved organ. There was a significant correlation between the intensity and extent of inflammation in each involved organ systems including the heart, lung, and lymph node, which are frequent sites of disease. This implies that, in daily clinical practice, the more extensively involved organs should be scrutinized for intensity of inflammation during image interpretation.

The study has several limitations. First, the present study is retrospective and the sample size was relatively small, partly because the focus of this study is patients with active cardiac sarcoidosis. Therefore, the results may not be generalizable to a larger population or to sarcoidosis patients without cardiac involvement. However, these findings are beneficial given the high morbidity and mortality of cardiac sarcoidosis. Second, we chose maintenance steroid dose at 6 months as a surrogate for treatment response, but this might not exactly reflect treatment response. However, patients’ response eventually reflect in their treatment, and treatment goal is to control cardiac sarcoidosis with medication including steroid of as low dose as achievable. Therefore, we believe it is justified to use steroid dose as a measure of treatment response. We were not able to incorporate objective follow-up findings such as PET/CT, pulmonary function test, and cardiac ultrasound into treatment response analysis because patient pool is heterogenous due to retrospective nature and not all patients had these follow-up studies. However, daily steroid use at the time of follow-up PET/CT might affect FDG activity in inflammatory foci in the heart and elsewhere due to elevations in blood glucose and insulin levels and compromise the results. Third, there was no reference standard for the diagnosis of cardiac sarcoidosis. None of the enrolled patients had positive endomyocardial biopsy; however, 10 of 16 patients had pathological evidence of sarcoidosis elsewhere and met the widely accepted diagnostic criteria established by the Japanese Ministry of Health and Welfare [18] or Heart Rhythm Society [19]. Six patients did not meet the criteria because these patients lacked extracardiac pathological confirmation of sarcoidosis, though three of them showed positive late gadolinium enhancement of cardiac MRI which was consistent with sarcoidosis involvement. These patients might have suffered from other cardiac inflammatory pathology though the diagnosis was made by our experienced sarcoidosis experts at the referring center. Fourth, patients underwent FDG PET/CT of the thorax and upper abdomen, not whole body coverage (from base of the skull to thigh), which is the standard protocol in oncology. The field of view from the thorax to the upper abdomen covers the most frequent sites of involvement in sarcoidosis, that said, there remains a possibility that disease involvement was underestimated.

## Conclusions

The maximum SUV across all involved organs of the chest and upper abdomen, not that of the heart alone, could be a predictor of treatment course of corticosteroid therapy in patients with presumed active cardiac sarcoidosis. This finding underscores the importance of evaluating extracardiac sites potentially, when evaluating patients with suspected sarcoidosis.

## Abbreviations

2D: Two dimensional
3D: Three dimensional
CT: Computed tomography
F: Fluorine
FDG: Fluorodeoxyglucose
MRI: Magnetic resonance imaging
PET: Positron emission tomography
SUV: Standard uptake value
TLG: Total lesion glycolysis
VOI: Volume of interest
